# Development of an efficient approach to boost fused deposition modeling (FDM) printing of felodipine-HPMC tablets for enhanced physical stability

**DOI:** 10.1016/j.ijpx.2025.100394

**Published:** 2025-09-12

**Authors:** Haixia Ren, Charles A. Laughton, Clive J. Roberts, Kam Loon Fow

**Affiliations:** a Department of Chemical and Environmental Engineering, University of Nottingham Ningbo China, Ningbo 315100, China; b School of Pharmacy, University of Nottingham, Nottingham NG7 2RD, UK; c School of Life Sciences, University of Nottingham, Nottingham NG7 2UH, UK

**Keywords:** Fused deposition modeling, Drug-polymer rheological study, Interaction parameter χ, Phase diagram, Physical stability.

## Abstract

Fused deposition modeling (FDM) is a widely investigated 3D printing technology for pharmaceutical applications because of its advantages, including easy access of equipment and cost-effectiveness. Despite its versatility, the FDM 3D printing process is multi-step, time-consuming and resource intensive, and requires a holistic selection of material and formulation design to achieve successful printing. The aim of this work is to develop an efficient approach to streamline FDM 3D tablet-printing processes, as well as to explore the factors that may influence the physical stability of the tablets produced. For this purpose, the polymer Affinisol™ HPMC HME 15LV and the model drug felodipine were selected for printing process evaluation, and the produced drug-polymer tablets were investigated for printing quality and physical stability. The approach began with a material-based rheological study to predict the processing temperature range for the hot melt extrusion process which was used to produce the required filament feedstock for FDM 3D printing. Flory-Huggins (F-H) theory and phase diagram were applied to guide the expected drug-polymer system physical stability with various ratios under different temperatures, suggesting that the system would be unstable when the weight fraction of felodipine is greater than 0.3. Based on these analyses, FDM 3D printed tablets with varying drug loads, printing temperatures, and infill densities were produced, and the long-term physical stability of the printed tablets under different conditions was monitored using XRD, DSC and polarized light microscopy. The results of the extrusion and printing process, as well as the observed long-term physical stability, were consistent with the findings from the predictions from the rheological study and theoretical evaluations. This suggests that an approach which combines rheological studies, theoretical evaluations and phase diagram construction could be applied as a pre-screen and optimization strategy, removing the need for full material characterization prior to FDM 3D printing of medicine. Such an approach significantly minimizes the amount of drug required, accelerates excipient selection, and optimizes the process for pharmaceutical manufacturing using FDM 3D printing. Furthermore, this methodology holds promise for broader application in other material-based FDM printing processes, offering a versatile framework for optimizing printing quality and stability across diverse systems.

## Graphical abstract

**Figure:**
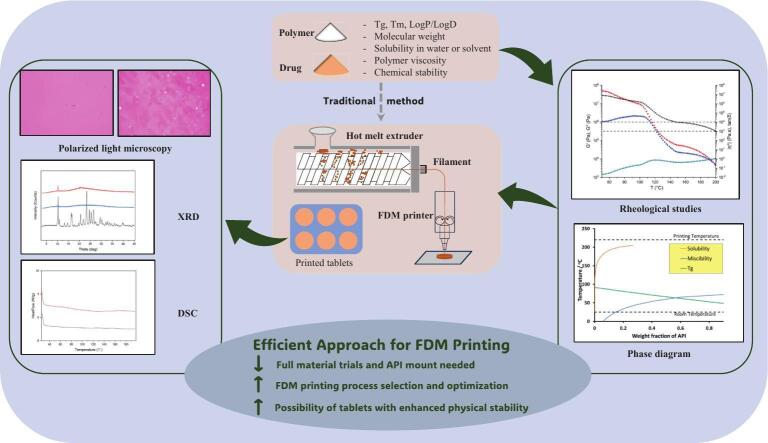

## 1 Introduction

Fused deposition modeling (FDM) is an additive fabrication technology that couples melt extrusion and three-dimensional (3D) printing (Bandari et al., 2021). This method allows the local control of material composition and microstructure, making it possible to create complex geometries that are difficult or impossible to achieve by conventional manufacturing processes (e.g., powder compression tableting) (Goyanes et al., 2015; Parulski et al., 2021). The FDM printing requires drug-loaded filaments which can be produced either by soaking the previously prepared filament into a drug solution or applying hot melt extrusion (HME) with physical mixtures (Melocchi et al., 2016; Sadia et al., 2016; Bandari et al., 2021; Henry et al., 2021).

Although FDM is a widely investigated 3D printing technology for pharmaceutical applications because of its advantages, including easy access of equipment and cost-effectiveness, several challenges have been repeatedly highlighted in those studies (Bernatoniene et al., 2025). Compared with a wide variety of material choices for medicinal products manufactured with conventional processes, FDM 3D printing has been restricted to a limited number of suitable pharmaceutical grade polymers that can be melted, extruded and printed (Melocchi et al., 2016; Sadia et al., 2016; Solanki et al., 2018a, 2018b; Pereira et al., 2020; Thakkar et al., 2020; Figueiredo et al., 2023). The properties and compositions of the materials used in the filaments required for printing can significantly affect the processability of the 3D printing. For successful filament production and subsequent 3D printing, it is necessary to elevate the temperatures to achieve rheological properties that are suitable for extrusion and printing. Hence, factors such as the melting point, viscosity, thermal stability of the materials, and the composition can impact both the extrudability (the ability to form filaments) and printability (the ability to form the desired object). Moreover, factors such as geometry design and printing parameters (e.g., infill density, printing temperature, etc.) can affect the desired dissolution and physicochemical stabilities of the medicinal products (Wang et al., 2023; Melocchi et al., 2016; Solanki et al., 2018a, 2018b; Sadia et al., 2016; Henry et al., 2021; (Horváth et al., 2025). Each step from filament fabrication to 3D printing requires precise control and a significant amount of material. Besides, the influence of environmental conditions like humidity on the moisture levels and mechanical characteristics of extruded filaments made from certain polymers has been noted (Figueiredo et al., 2023). This can significantly impact their suitability for printing. Therefore, the FDM 3D printing process is relatively complex and time-consuming because it requires a deep understanding of the materials, technology and design involved.

To date, there are limited reports in the literature outlining a structured method to streamline and accelerate solid-dosage form printing from HME fabrication of drug-loaded filaments to FDM printing. Several publications have been reported for streamlining the process of FDM 3D printing or predicting the performance of fabricated products (e.g., tensile strength, dissolution) by using computational AI model; however, this approach requires the use of extensive data from massive experiments (Elbadawi et al., 2020; Huang et al., 2024; Kharate et al., 2024; Panico et al., 2025; Wei et al., 2024; Wikło et al., 2025). Given these premises, the aim of this study is to develop a structured method which can help minimize the amount of drug required, accelerate excipient selection, and optimize the process for pharmaceutical manufacturing using FDM 3D printing.

For HME for pharmaceutical application, it has been reported that conducting a comprehensive evaluation of various key factors related to the model drug and polymer can be beneficial for formulation design and process optimization with the HME process (Censi et al., 2018). These factors include the chemical and thermal degradation of drug-polymer mixtures, the physicochemical stability of products, potential drug-polymer interactions, the miscibility/solubility of the drug-polymer system, and rheological properties. Considering that both the extrusion process and subsequent printing process in FDM 3D printing involve heating and shearing, and the printing process is essentially a melt extrusion process, there is a high potential for FDM 3D printing to benefit from a similar methodology used in the design of the HME process.

Numerous studies have highlighted the applications of rheological data in the design and further process development of solid dispersions via HME (Gupta et al., 2014; Yang et al., 2016). The practical application of rheology in guiding the optimal selection of HME processing temperature for the preparation of copovidone and nifedipine amorphous solid dispersion has been demonstrated. Recently in-depth analyses have been conducted of rheological investigations for 3D printing (Zong et al., 2023; Sun et al., 2023), and it has been recommended that a complex viscosity of less than 8 kPa⋅s is desirable for sufficient material flow from a heated FDM 3D printer nozzle (Isreb et al., 2019). Furthermore, some studies have leveraged rheological studies to investigate the influence of viscoelastic behavior on printability (Calafel et al., 2020, Pinho et al., 2021). Despite these advances, exploration into the application of rheological studies for both HME process and FDM 3D printing remains an area of ongoing study.

The drug-polymer system can be viewed as a drug dispersed into a polymeric matrix, where the drug might exist as undissolved particles, a solid solution, or both. These different physical states of drug-polymer system will subsequently impact the product stability, crystallisation tendency and subsequent drug dissolution. The likelihood of physical instability and associated drug precipitation during dissolution can be diminished if the drug and polymer establish stable or significant interactions. These interactions are intrinsically linked to the drug and polymers' solubility and miscibility (Li et al., 2014), which are related with the drug-polymer composition and manufacturing process (Shi et al., 2020). Various methods can be used to estimate the miscibility of a drug and polymer. One method is the use of Hansen solubility parameters (HSPs), which describe the total cohesive energy as a sum of partial solubility parameters. These include nonpolar atomic (dispersion) interactions (δ_d_), dipole-dipole molecular interactions (δ_p_), and hydrogen bonding (δ_h_) (Lakshman et al., 2020). The difference in HSP between two components can provide an indication of their miscibility. Additionally, the Flory-Huggins (F-H) interaction parameter (χ) can provide a quantitative description of miscibility from a thermodynamic perspective of mixing, using a melting point depression approach (Hu et al., 2022; Potter et al., 2018). Drug−polymer thermodynamic phase diagrams based on the F-H theory have been used to predict the physical stability of amorphous systems. The phase diagram can clearly identify metastable and unstable drug-polymer concentrations for each system at varying temperatures (Tian et al., 2013). This information can help guide the selection of the appropriate drug-polymer composition and predict the stability or instability potentials at different temperatures for a given composition.

Hence, this study is to develop a structured method that combines the assessment of drug-polymer physical-chemical properties with miscibility or solubility through phase diagram construction and rheological properties before the FDM 3D printing. This approach may reduce the processing exploration time, streamline the workflow, and accelerate the drug printing from HME fabrication of drug-loaded filaments to FDM 3D printing. Additionally, this study will also explore the key factors (e.g., drug loads, printing temperatures, and infill densities) that may affect the physical stability of printed products, potentially leading to the verification and optimization of the established approach. Felodipine (FEL) is selected as the model drug in this study due to its good glass-forming property and thermostability with melting point of around 145 °C (Kim et al., 2006; Guo et al., 2020). Affinisol™ HPMC HME 15LV, a variant of hydroxypropyl methylcellulose (HPMC), is chosen as the polymer in this study. Due to its unique polymer substitution, 15LV grade has a lower glass transition temperature (T_g_) at around 90 °C (Khatri et al., 2018) and a lower melt viscosity, which is suitable for HME process between 135 and 190 °C (Svoboda et al., 2023). To better explore the different impact factors which were highlighted above, the plasticizer was not investigated in this study.

Furthermore, literature on the applications of FEL and HPMC in FDM 3D printing have been reviewed, offering a comprehensive analysis of their suitability and potential benefits for this technology. Several investigations have explored various polymers to enhance printability and control drug release from FEL (Iovanov, 2023, Iovanov, 2024; Mora-Castaño et al., 2024; Solanki et al., 2018a, 2018b). One such study (Mora-Castaño et al., 2024) employed solubility parameters and molecular dynamics simulations to predict FEL-HPMC miscibility, which did not cover the printing process prediction. This study aimed to build upon more pre-screening efforts, establish one structured approach, facilitate the selection of drug-polymer components and optimize the FDM printing process of tablets for enhanced physical stability while minimizing API consumption.

## 2 Materials and methods

### 2.1 Materials

The drug material (FEL, lot NCL/XIX/APL/15/003) was supplied by Anek Prayog Pvt. Ltd. (India). The HPMC polymer (Affinisol™ HPMC HME 15LV, lot F293G2A002) was supplied by International Flavors & Fragrances (China).

### 2.2 Methods

#### 2.2.1 Methodology

**Fig. 1:**
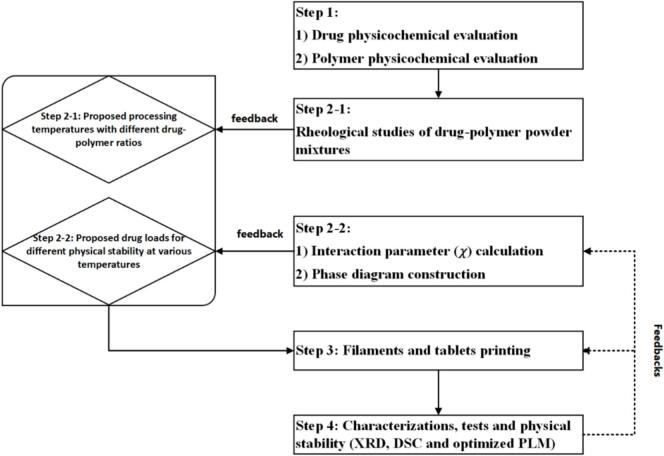
Methodology of streamlining formulation and process development for FDM 3D printing of tablets.

The elevated temperatures and shear rates utilized in FDM 3D printing could potentially lead to the degradation of the drug and excipients. Therefore, it is crucial to assess the physicochemical properties of FEL drug and HPMC polymer to ensure optimal thermostability. Moreover, the desired dissolution profiles may limit the selection of excipients, as those designed for enteric or delayed release may not be suitable for immediate release products. The initial step hence involves a thorough evaluation of the physicochemical properties of the drug and polymer. The second step includes conducting rheological studies of the drug-polymer powder mixtures, determining the interaction parameter χ using the ball milling method and constructing a phase diagram (Tian et al., 2013). These steps are expected to provide preliminary formulation and processing data to aid the FDM 3D printing process. The produced tablets are then characterized and their physical stability is assessed. The findings from the manufacturing process and characterizations can provide further feedbacks for the evaluations conducted in steps 2.2 and 3. The proposed methodology is summarized in Fig. 1.

#### 2.2.2 Rheological study of FEL-HPMC powder mixture

Rheological measurements were undertaken using a rheometer (HAAKE MARS 60, Thermo Fisher Scientific, Germany) with the HAAKE Rheowin software (versions 4.93.0003), equipped with a 20 mm parallel plate. A binary formulation of FEL and HPMC with different weight ratios (*w*/w: 0:10, 1:9, 3:7, 1:1) was physically mixed separately. With a spatula, a disk-shaped mixture layer of each formulation was formed (diameter: 20 mm, thickness: approx. 2 mm) with loading temperature 130 °C. An oscillatory strain sweep was conducted to determine the linear viscoelastic region (LVR) at a high printing temperature of 200 °C with an amplitude strain of 0.1 % and a fixed frequency of 0.1 rad.s^−1^. The sample was heated from 50 to 200 °C at a heating rate of 4 °C min^− 1^.

#### 2.2.3 Ball milling of FEL-HPMC powder mixture for phase diagram construction

Samples ranging from 0.8 to 1.0 g with varying drug and polymer compositions were initially mixed using a vortex mixer (JL-D, Xinlan, China) for 3 min and then processed in a ball mill mixer (model MM200, Retsch, Germany). The FEL and polymer powder mixture were ground using a single stainless-steel ball (diameter: 8.5 mm) at a frequency of 25 Hz. Each milling cycle lasted 2 min, followed by a 2 min interval, and the procedure was repeated for a total of 12 cycles for the determination optimum milling conditions using subsequent analysis by differential scanning calorimetry (DSC) method as detailed method in section 2.2.5. The phase diagram was constructed using F-H theory to forecast the solubility and miscibility of the drug within polymeric systems. These predictions were based on varying drug-polymer ratios and temperatures, which are associated with physical stability (Lin and Huang, 2010; Sun et al., 2010). The variation in Gibb's free energy of mixing function (ΔG_mix_) can be expressed as a function of the F−-H interaction parameter, χ, as depicted in Eq. 1:

(1) $$\frac{\Delta G_{\mathrm{mix}}}{\mathrm{RT}}=\phi_{\mathrm{drug}}\ln\phi_{\mathrm{drug}}+\frac{\left(1-\phi_{\mathrm{drug}}\right)}{m}\ln\left(1-\phi_{\mathrm{drug}}\right)+\phi_{\mathrm{drug}}\left(1-\phi_{\mathrm{drug}}\right)\chi$$

where $\phi_{\text{drug}}$ is the volume fraction of the drug in the polymer, m is the molar volume ratio of polymer to drug, χ is the F-H drug-polymer interaction parameter, R is the molar gas constant, and T is the temperature. The data on melting point depression gathered from DSC can be utilized to predict the drug-polymer interaction parameter by applying the subsequent Eq. 2 (Paudel et al., 2010):

(2) $$\left(\frac{1}{T_{m}^{\mathrm{mix}}}-\frac{1}{T_{m}^{\mathrm{pure}}}\right)\frac{\mathrm{\Delta H}}{-R}-\ln\phi_{\mathrm{drug}}-\left(1-\frac{1}{m}\right)\phi_{\mathrm{polymer}}=\chi_{\mathrm{drug}-\mathrm{polymer}}\phi_{\mathrm{polymer}}^{2}$$

where $\phi_{\text{drug}}$ is the volume fraction of the drug, $\phi_{\text{polymer}}\;$is the volume fraction of the polymer,$T_{m}^{\mathrm{mix}}$ and $T_{m}^{\mathrm{pure}}$ are the melting points of drug-polymer mixture and the pure drug, respectively, R is the gas constant, and ΔH is the heat of fusion of the drug (84.55 J/g). The gradient of the plot of Eq. 2 is the interaction parameter χ at the melting point of the pure drug.

The interaction parameter χ at room temperature can be calculated based on the Hansen solubility parameters (Lindvig et al., 2002) using Eq. 3:

(3) $$\chi_{\mathrm{drug}-\mathrm{polymer}}=\frac{V_{1}}{\mathrm{RT}}\left({\left(\delta_{1,d}-\delta_{2,d}\right)}^{2}+0.25{\left(\delta_{1,p}-\delta_{2,p}\right)}^{2}+0.25{\left(\delta_{1,\mathrm{hb}}-\delta_{2,\mathrm{hb}}\right)}^{2}\right)$$

where $V_{1}$ is the molar volume (cm^3^ mol^−1^) of drug, T is temperature, $\delta_{1,d}$, $\delta_{1,p}$, $\delta_{1,\mathrm{hb}}$ are contributions from non-polar (dispersion) forces (d), polar forces (p) and hydrogen-bonding (hb) effects related to the drug. Whilst $\delta_{2,d}$, $\delta_{2,p}$, $\delta_{2,\mathrm{hb}}$ are the corresponding contributions related to the polymer.

The χ values at different temperatures can be determined by the Eq. 4, which is projected from the interaction parameter χ at room temperature and melting point of the pure drug. Wherein any dependency on composition is neglected, *A* is the value of the temperature-independent term, *B* is the value of the temperature dependent term (Rubinstein and Colby, 2003).

(4) $$\chi_{\mathrm{drug}-\mathrm{polymer}}=A+\frac{B}{T}$$

Furthermore, the maximum drug−polymer miscibility boundary (spinodal curve) can be calculated by taking the second derivative of the free energy of mixing (Eq. 1) equivalent to 0 and identifying Φ as a function of χ.

#### 2.2.4 HME for filaments fabrication and FDM 3D printing for tablets printing

The FEL-HMPC mixtures, with a drug concentration ranging from 0 to 50 % *w*/w, were adequately blended before charging to the hot melt extruder. A 11 mm co-rotating twin screw extruder (Thermo Fisher Scientific, Germany) was used to prepare the filaments with standard fixed screw design. A die diameter of 1.50 mm was selected for HME to produce filament which can be fed into the commercial FDM 3D printer (K5, SGON, China, suitable for filaments with diameter 1.75 mm). The 0.4 mm nozzle diameter of the printer was selected. The FEL-loaded filaments were extruded under various barrel temperatures (ranging from 110 to 195 °C) with a screw speed of 10–20 rpm. The filaments were cooled using an air conveyor (Thermo Fisher Scientific, Germany) at room temperature based on a nominal analogue scale, and the detailed parameters are summarized in Table 1. In this study, the holding time for filaments was limited to no more than a week to mitigate the influence of environmental humidity on their mechanical properties and water content before proceeding to the downstream FDM printing step. Furthermore, the filaments were stored in low density polyethylene resin bag sealed with crimps at room temperature with desiccants. All the specified designs and controls were derived from prior small-scale feasibility studies.

**Table 1:** Summary of formulations and extrusion parameters.

Filament	FEL: HPMC (*w*/w)	HME temperature (°C)			Remarks
		zone 1	zone 2	zone 3–8	
F1–1	0: 100	135	155	195	Control
F2–1	10: 90	110	140	160	Low processing temperature
F2–2	10: 90	110	140	180	High processing temperature
F3–1	30: 70	110	140	140	Low processing temperature
F3–2	30: 70	110	160	160	High processing temperature
F4–1	50: 50	125	160	160	Low processing temperature
F4–2	50: 50	135	180	180	High processing temperature

The model tablets (diameter: 6 mm, thickness: 2.5 mm) were designed using SketchUp software (Pro 18.0) and converted to .gcode files using CURA software (version 4.10). The tablets were printed with a printing speed of 40 mm s^−1^ and bottom/top thickness of 0.4 mm. Layer height of 0.2 mm and shell thickness of 0.4 mm were applied for all tablets printing. The processing parameters (printing and bed temperature, infill density) are tabulated in Table 2.

**Table 2:** Printing parameters used in the fabrication of FDM 3D printed tablets.

Tablet name	Printing temperature (°C)	Bed temperature (°C)	Infill Density (%)
*Tablet from filament F1–1 as control*			
T1–1-20 %	190	100	20
T1–1-80 %	190	100	80

*Tablet from filament F2–1 (FEL-HPMC: 1:9 w/w) with low HME and printing temperature*			
T2–1-20 %	170	70	20
T2–1-80 %	170	70	80

*Tablet from filament F2–2 (FEL-HPMC: 1:9 w/w) with high HME and printing temperature*			
T2–2-20 %	190	100	20
T2–2-80 %	190	100	80

*Tablet from filament F3–1 (FEL-HPMC: 3:7 w/w) with low HME and printing temperature*			
T3–1-20 %	170	70	20
T3–1-80 %	170	70	80

*Tablet from filament F3–2 (FEL-HPMC: 3:7 w/w) with high HME and printing temperature*			
T3–2-20 %	190	100	20
T3–2-80 %	190	100	80

#### 2.2.5 UV method for the drug content of printed tablets

The printed tablet (n = 3) was transferred to a 100-mL volumetric flask, and 20 mL of 1 % (*w*/*v*) polysorbate 80 in water was added to the flask. The mixture was sonicated for 5 min. Methanol (20 mL) was added under sonication to dissolve the drug. The mixture was cooled to room temperature anddiluted with 1 % (*w*/*v*) polysorbate 80 in water to volume Drug content was analysed using a UV-VIS spectrophotometer (UV-2450; Shimadzu, Japan) with 263 nm wavelength. The drug content was calculated based on the validated calibration curve (A = 0.017C + 0.0057, R^2^ = 1). The result is presented as a percentage ratio of the measured drug content to the expected weight of drug.

#### 2.2.6 DSC for crystallinity characterization

DSC analysis was conducted using a Q2000 DSC (TA, America) under nitrogen flow of 50 mL min- 1. Samples (FEL-HPMC powder mixtures or produced tablets) between 5 and 10 mg were analysed using aluminium crimped pans with punctured lids, and an empty aluminium pan with lid was used as reference.

Two cycles of heating were applied, a heating rate of 10 °C min^-1^ was set between 25 °C and 100 °C, and held for 2 min to eliminate moisture interference, then cooled back to 25 °C.The second cycle of heating was initiated with a heating rate of 10 °C min^-1^ to 200 °C. Only the second cycle result is shown in the data.

#### 2.2.7 Powder X-ray diffraction (XRD) for crystallinity characterization

XRD patterns were acquired using a Rigaku Ultima IV diffractometer (Japan) with Cu Kα radiation (40 kV, 40 mA). The data collection spanned a 2θ range of 3–45°, with a scan rate of 20° min^−1^ at ambient temperature. The Origin software (Jade, version 6.2.9200(2)) was employed to display the data, showcasing peak positions at 2θ and X-ray intensity with counts.

#### 2.2.8 Polarized Light Microscopy (PLM) for crystallinity characterization

The tablet fragments were placed on a glass slide and drops of water were added to remove the interference of birefringence from the polymer. A scraper was gently applied to mix the materials and water till a gel formed slowly. The semi-solids on the glass slide were used for PLM (DM750P, Leica, Germany) characterization with a 530 nm compensator using software FCSnap (version x64, 1.1.21162.20220720).

#### 2.2.9 Physical stability of the produced tablets

All printed tablets in open containers were exposed to accelerated stability test conditions of 40 °C and 75 % relative humidity (RH). Given the information provided in the literature regarding the impact of environmental conditions on the physical stability of amorphous dispersions (Tian et al., 2013), a 5 % low relative humidity (RH) environment was chosen to minimize the effects of moisture on products based on cellulose excipients. Consequently, room temperature 25 °C with 5 % low RH was selected. The physical stability of each tablet was evaluated at different timepoints up to 495 days by determining its crystallinity with XRD, DSC and PLM method, which refers to physical stability. The drug content was calculated at the last timepoint 495 days with UV method.

## 3 Results and discussion

### 3.1 Rheological study of FEL-HPMC powder mixture

**Fig. 2:**
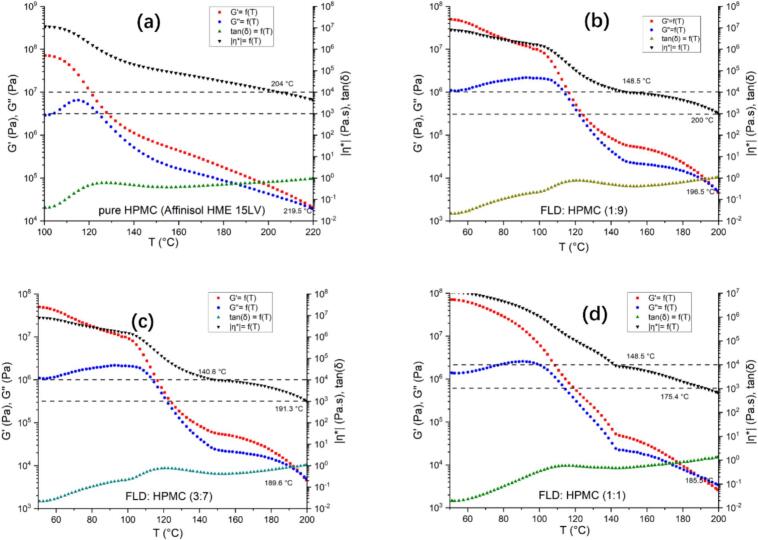
Storage modulus (G′), loss modulus (G″), complex viscosity (η*), and loss tangent (tan δ) vs temperature taken at constant corresponding frequencies of 0.1 rad s^−1^ for the FEL-HPMC powder mixtures (a): HPMC; (b): 1:9 (*w*/w); (c): 3:7 (w/w); (d): 1:1 (w/w).

The measured values of storage modulus (G′), loss modulus (G″), complex viscosity (η*), and loss tangent (tan δ) as a function of temperature for samples of FEL-HPMC powder mixture with various weight ratios (0:10, 1:9, 3:7, 1:1) are depicted in Fig. 2. These values were used to determine the HME and following 3D printing processing temperatures. Before starting the rheological studies, air bubbles were expelled from the samples while preserving the drug crystallinity at a loading temperature of 130 °C (Yang et al., 2016, Yang et al., 2016b). G′ measures the elastic reaction, while G″ corresponds to the measure of the viscous reaction. If G″ surpasses G′ across all examined frequencies, the materials exhibit a predominantly viscous character above the crossover temperature (G′=G″), where the tan δ equals 1. Prior reports suggest that for successful melt extrusion, the complex viscosity η* of the material should ideally be between 1000 and 10,000 Pa.s at an angular frequency of 0.1 rad.s^−1^ (Gupta et al., 2014; Solanki et al., 2018a, 2018b). Therefore, the processing temperature should meet two conditions. it should be higher than the crossover temperature (G′ = G″ or tan δ = 1)and allow η* to be within 1000–10,000 Pa s. However, HPMC is reported to be sensitive to the shear rate applied, which can significantly reduce its viscosity during extrusion. This allows HPMC to be processed at temperatures lower than the typical η* range of 1000 to 10,000 Pa s. generated by an oscillatory rheometer (Gupta et al., 2014). Additionally, the shear stress in FDM 3D printing is different from standard HME, potentially resulting in different processing temperature ranges. Nonetheless, initial assessments of shear rate impacts on viscosity can be made using rheological studies.

For HPMC polymer in Fig. 2(a), the complex viscosity η* reaches 10,000 Pa⋅s at 204 °C, with the crossover temperature (G′ = G″) at 219.5 °C. The HME processing range is reported from 135 °C to 190 °C on the supplier IFF website (Svoboda et al., 2023). Hence, the processing temperature was set at 135–190 °C for HME and 190 °C for 3D printing due to its lower shear rate. Although Fig. 2(b) and (c) are generated with different weight ratio, the results are similar. For the FEL-HPMC powder mixture with a weight ratio of 1:9 in Fig. 2(b), the η* is 10,000 Pa⋅s at 148.5 °C and decreases to 1000 Pa⋅s at 200 °C. The crossover temperature (G′ = G″) is at 196.5 °C. Considering the shear rate difference in melt extrusion and 3D printing, as well as the sensitivity of HPMC to the shear rate, the starting temperature was set at 195 °C for 3D printing and 148.5–200.0 °C for extrusion due to the higher shear rate. For the mixture with a weight ratio of 3:7 in Fig. 2(c) similar considerations apply. The starting point for 3D printing was set at 190 °C, close to the crossover temperature, with a range of 140.6–191.3 °C for extrusion. For the mixture with a weight ratio of 1:1 in Fig. 2(d), η* decreased dramatically from 10,000 to 1000 Pa⋅s within 149–175 °C, and the crossover temperature is 185.5 °C. The melt extrusion temperature range is proposed at 148.5–185.5 °C. However, low viscosity above 175.4 °C may lead to poor extrudability.

### 3.2 Phase diagram construction based on the FEL-HMPC powder mixture

A pure drug melts at the temperature where the crystalline drug's chemical potential matches that of the melted drug. If the drug is miscible with a polymer and dissolves in it, the chemical potential of the drug in the polymer becomes lower than in its pure melted state. This occurrence results in melting point depression of drug crystals within the polymer matrix (Lin and Huang, 2010; Tian et al., 2013).

**Fig. 3:**
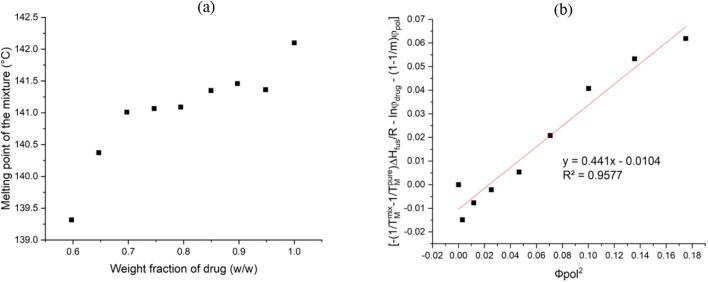
(a) DSC results of ball milled FEL-HPMC powder mixtures measured at a heating rate of 10 °C min^−*1*^. (b) F − H interaction plot used to determine the F-H interaction parameter close to the melting point of the drug*.*

In this case, a ball milling method has been employed to determine the depressed melting point (endset point). The endset point was calculated as the point where the endothermic trace intersected with the baseline after melting. Ball milling may increase the amorphous content of the drug, enhance the drug-polymer interaction, decrease its chemical potential, and consequently depress the drug melting (Tian et al., 2013; Sun et al., 2010). Given that the endset point of melting can be lowered with an increase in milling frequency and duration, it's crucial to identify the best milling conditions to optimize the potential interaction between the two components. The appropriate utilization of ball milling within a suitable time scale could potentially enhance the drug-polymer interaction at the molecular level and provide more precise data on the depression of the melting point. The optimum milling duration was found to be 32 min, which included 8 cycles of 2 min of milling and 2 min interval. Beyond the 32 min mark, no further melting point depression was observed. Fig. 3(a) shows the DSC results obtained for the ball milled samples containing different weight fractions of drug. The F−H interaction parameter χ can be calculated by Eq. 2. A plot of (1/ T_m_^mix^ – 1/T_m_^pure^) × (ΔH_fus_/−R) – ln(ϕ_drug)_ – (1–1/m) ϕ_polymer_ versus ϕ_polymer_^2^, as shown in Fig. 3(b), is used to calculate the F-H interaction parameter close to the melting point of the drug. An interaction parameter with a value of – 0.0104 (r^2^ = 0.9577) was obtained, and the χ value was calculated as 0.441 at the melting point of felodipine. This slightly positive value suggests that the interactions between this HPMC polymer and felodipine are slightly stronger than the interaction within the same species. While immiscible systems usually exhibit large positive interaction values, it is noteworthy that the interaction parameter values obtained here are close to zero.

To further investigate the interaction parameter χ values at different temperatures, χ for FEL-HPMC system at 25 °C was calculated using the solubility parameter method (Eq. 3) based on the group contribution summary in Table 3. χ at 25 °C was calculated to be 4.11.

**Table 3:** Group contributions summary for Hansen Solubility Parameter.

	Hansen Solubility Parameter (Dispersion) (J/cc)^1/2^	Hansen Solubility Parameter (Polar) (J/cc)^1/2^	Hansen Solubility Parameter (Hydrogen Bonding) (J/cc)^1/2^
FEL	18.0	11.9	12.3
HPMC	22.4	5.2	8.4

**Fig. 4:**
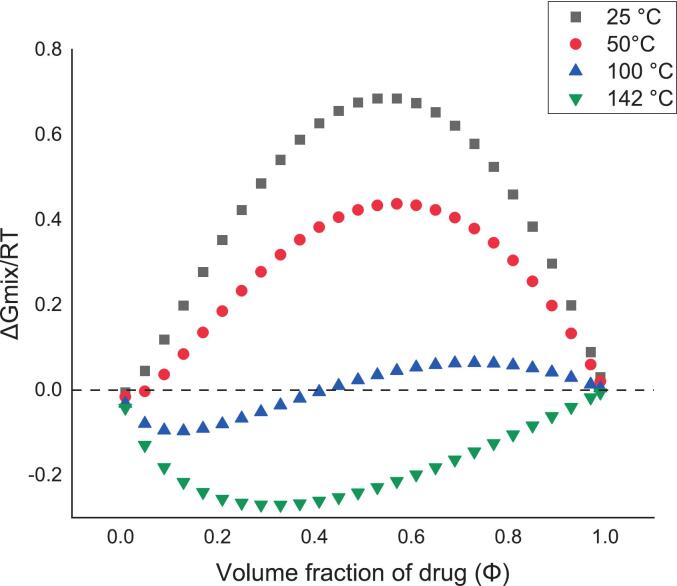
Plot of *ΔG*_*mix*_ /RT as a function of drug volume fraction for felodipine and HPMC at temperatures of 25, 50, 100, and 142 °C.

The interaction parameter χ at 25 °C (4.11 at 298 K) was positive and larger than that at the drug melting point (0.441 at 415.25 K). This suggests a reduced miscibility for the drug-polymer system with decreased temperature. F-H interaction constants *A* and *B* were determined according to Eq. 4, using interaction parameter χ, calculated at 25 °C and melting point of the drug. The values “*A*” and “B” were 8.88 and 3872.16, respectively. The values of χ at different temperatures can be obtained and the changes in Gibb's free energy as a function of drug volume fraction can be plotted using Eq. 1. The details are described in Fig. 4. At elevated temperature, the ΔG_mix_ starts to decrease. When the ΔG_mix_ becomes negative and convex, it signifies the formation of homogeneous mixtures (Tian et al., 2013). The mixtures are thermodynamically stable across all compositions of drug and polymer at around 140 °C, and the mixtures are thermodynamically stable with drug volume fraction no more than 0.4 at 100 °C. With lower processing temperature, the mixtures may be less thermodynamically stable.

**Fig. 5:**
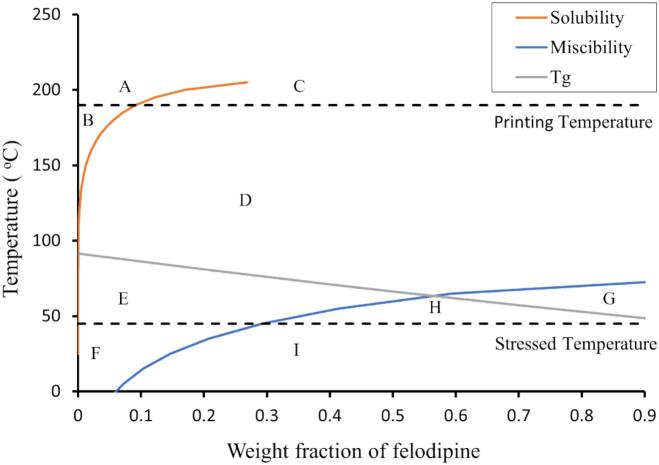
Binary phase diagram for felodipine and HPMC. From left to right, top to bottom: Region ‘A’ and ‘B’: stable state; Region ‘C’ to ‘E’: metastable state; Region ‘G’ to ‘I’: unstable state.

Utilizing the F-H theory, the phase diagram was constructed and is shown in Fig. 5. The solubility boundary curve indicates the fraction of crystalline felodipine dissolving into the HPMC polymer matrix as a function of both processing temperature and composition. If the system's temperature drops below the solubility boundary, amorphous phase separation may take place prior to drug crystallisation. The miscibility boundary curve, also known as the spinodal curve, represents a change in the mechanism of amorphous phase separation (Lin and Huang, 2010; Tian et al., 2013). In this scenario, the drug exists in a metastable state (zone C – F in Fig. 5) within the polymer between the solubility and miscibility boundaries and in an unstable state below the miscibility boundary (zone G – I in Fig. 5).

The glass transition T_g_ curve shown in Fig. 5 was predicted according to Eq. 5:

(5) $$T_{g}=1/\left(X/T_{g1}+\left(1-X\right)/T_{g2}\right)$$

where the T_g1_ and T_g2_ are the temperatures related to felodipine (317.55 K) and HPMC (364.67 K), X is the weight fraction of felodipine in this case. The predicted T_g_ of mixtures serves as an indicator of stability. When the system is below the T_g_, phase separation is thermodynamically advantageous. However, theoretically, the molecular mobility will decrease dramatically below the T_g_, and the properties of system could present significant kinetic obstacles to recrystallization, leading to the stabilization of the system over time. Hence, the system may have better physical stability if the drug-polymer system formation temperature is high, and the storage temperature is low.

With the constructed phase diagram (Fig. 5), the physical stability of the produced amorphous system can be predicted under different temperatures or compositions. In this case, when the felodipine weight fraction in the system is less than 0.29 (equivalent to a 31 % drug load, *w*/w), the system remains stable (in zone A and B) or metastable (in zone C and D in Fig. 5) during the HME and printing process. However, it enters the metastable zone E as the system cools to the stressed storage temperature 45 °C. In contrast, if the felodipine weight fraction exceeds 0.29, the system transitions into unstable, entering either zone H or G at the stressed storage temperature of 45 °C, or zone I at room temperature (25 °C). This suggests that a felodipine weight fraction of not more than 0.29 is recommended for system physical stability. If the felodipine weight fraction is increased further, the unstable zone of system becomes wider in terms of temperature.

### 3.3 Filaments for printing and tablets from FDM 3D printing

Table 4 provides a summary of the data on fabricated filaments and FDM 3D printed tablets. For filaments F4–1 and F4–2, which contain FEL-HPMC (*w*/w 50:50) as described in Table 1, the diameter could not be consistently controlled, making them unsuitable for feeding into the subsequent FDM printer. The high variability of filament diameter (F4–1, F4–2) may be attributed to the significant reduction in complex viscosity η* during the extrusion process with elevated temperature as indicated in Fig. 2(d).

**Table 4:** Summary of fabricated filament and FDM 3D printed tablets.

Filament	Extrudability	Tablet	Printability
F1–1	Extrudable^1^	T1-1-20 %	Printable^3^
		T1-1-80 %	Printable^3^
F2-1	Extrudable^1^	T2-1-20 %	Printable^3^
		T2-1-80 %	Printable^3^
F2-2	Extrudable^1^	T2-2-20 %	Printable^3^
		T2-2-80 %	Printable^3^
F3-1	Extrudable^1^	T3-1-20 %	Printable^3^
		T3-1-80 %	Printable^3^
F3-2	Extrudable^1^	T3-2-20 %	Printable^3^
		T3-2-80 %	Printable^3^
F4-1	Extrudable^2^	T4-1-20 %	Failed
		T4–1-80 %	Failed
F4–2	Extrudable^2^	T4-2-20 %	Failed
		T4–2-80 %	Failed

1 The filaments can be extruded through the nozzle with the anticipated diameter.

2 The filaments can be extruded through the nozzle, but the variability of diameter is high.

3 The tablets can be printed with designed shape; the surface is uniform and smooth with no collapse structure.

From the previous prediction from the rheological study, the proposed extrusion temperature was 148.5–185.5 °C, and the actual processing range was within the scope. For other filaments containing HPMC to FEL-HPMC (w/w 3:7), the extrudability and printability were good. The integrity of the printed tablets was maintained with no visible cracks or deformities. The tablets exhibited a uniform and smooth surface. Their color varies from light to dull pale yellow, tending to become duller pale yellow as drug loading decreases or processing temperature increases. The tables have a round shape. The actual extrusion (160–180 °C, zone 3–8) and printing (170–190 °C, nozzle area) temperatures for filament containing FEL-HPMC (w/w 1:9) were consistent with the rheological result shown in Fig. 2(b).The actual extrusion (140–160 °C, zone 3–8) and printing (170–190 °C, nozzle area) temperatures for filament containing FEL-HPMC (w/w 3:7) were consistent with the rheological result showed in Fig. 2(c). Hence, the rheological study is validated as an approach to provide insight into predicting the extrudability and printability of materials and selecting suitable temperature conditions, which might contribute to streamlining the fabrication FDM 3D printing process. For HPMC in Fig. 2(a), the filaments cannot be extruded properly at temperatures below 195 °C. The printing temperature is 190 °C, which is also below 204 °C. Given that HPMC is known to be highly sensitive to applied shear rates (Gupta et al., 2014), it's anticipated that the temperature ranges would be broader in the HME process with the high shear rates experienced. This has been demonstrated in filament fabrications where the extrusion temperature could be below the crossover temperature (G′ = G″). Therefore, for more accurate predictions, it is crucial to have a thorough understanding of material properties. In this case, the processing range of HPMC is much lower than the prediction outcome of rheology study, because this material is sensitive to the shear rate applied. The printing temperature is higher than the extrusion temperature due to the less shear rate applied in 3D printing.

### 3.4 Characterization of produced FDM 3D printed tablets and physical stability

All produced tablets were characterized at the initial timepoint for the drug content, solid-state form for the presence of crystallinity using XRD, DSC and PLM. For physical stability, all tablets were taken out at different timepoints to be characterized by XRD, DSC and PLM, and the drug content was calculated in the last timepoint.

#### 3.4.1 Drug content of printed tablets

The drug contents of the printed tablets were evaluated (Table 5). The results indicated that the drug may not mix effectively with the polymer during the preparation of formulations T3–1-20 % and T3–1-80 %, as both tablet formulations originated from the same blend, and their initial drug content was approximately 91 %. The initial drug content for other printed tablets was close to or exceeded 97 %. Furthermore, the results from the final timepoint demonstrated that the printed tablets remained chemically stable at 40 °C/75 % RH after 495 days. Samples stored at 25 °C/5 % RH were not tested, as stability under harsher conditions had already been confirmed.

**Table 5:** Summary of drug content of FDM 3D printed tablets.

Tablet Name	Drug content ± SD (%)	Drug content ± SD (%)
	Timepoint Initial	Timepoint 495 d (40 °C/75 %RH)
T2-1-20 %	97.21 ± 0.39 (*n* = 3)	97.74 ± 0.96 (n = 3)
T2-1-80 %	97.53 ± 0.79 (n = 3)	97.35 ± 1.02 (n = 3)
T2-2-20 %	97.51 ± 0.08 (n = 3)	97.01 ± 0.73 (n = 3)
T2-2-80 %	97.59 ± 1.10 (n = 3)	97.19 ± 1.70 (n = 3)
T3-1-20 %	91.30 ± 1.48 (n = 3)	91.97 ± 0.86 (n = 3)
T3-1-80 %	90.64 ± 0.79 (n = 3)	91.42 ± 0.84 (n = 3)
T3-2-20 %	98.16 ± 1.62 (n = 3)	97.18 ± 1.32 (n = 3)
T3-2-80 %	96.92 ± 1.00 (n = 3)	97.08 ± 1.92 (n = 3)

#### 3.4.2 PLM method development

**Fig. 6:**
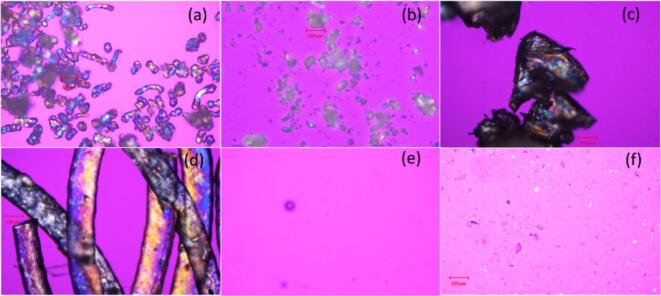
PLM (530 nm compensator) comparison using method with pre-treatment or no pre-treatment. From left to right, top to bottom: (a) AffinisolTM HPMC HME 15LV (no pre-treatment) (b) Crystalline felodipine (no pre-treatment) (c) filament with 10 % drug loads (*w*/w) at 0 d (no pre-treatment) (d) tablet with 10 % drug loads (w/w) at 0 d (no pre-treatment) (e) tablet with 10 % drug loads (w/w) at day 0 (pre-treatment) (f) tablet with 10 % drug loads (w/w) at 495 d in 40 °C/75 %RH storage condition (pre-treatment).

During the PLM method development, the traces of birefringence can be observed in Affinisol™ based samples - amorphous polymer (a), amorphous filament with 10 % drug loads (c) and 3D printed amorphous tablet with 10 % drug loads (d) (Fig. 6) when characterizing these samples without any pre-treatment with water, interfering with signals from crystalline materials. To remove the interference of birefringence, the materials were pre-treated with water for crystalline characterization for the PLM method, and no traces of birefringence can be observed for the 3D printed amorphous tablet with 10 % drug loads in Fig. 6(e). However, for drugs with higher water solubility, this pre-treatment could result in partial dissolution of recrystallized drug form, or changes in the amorphous fraction, potentially compromising the validity of the characterization.

**Fig. 7:**
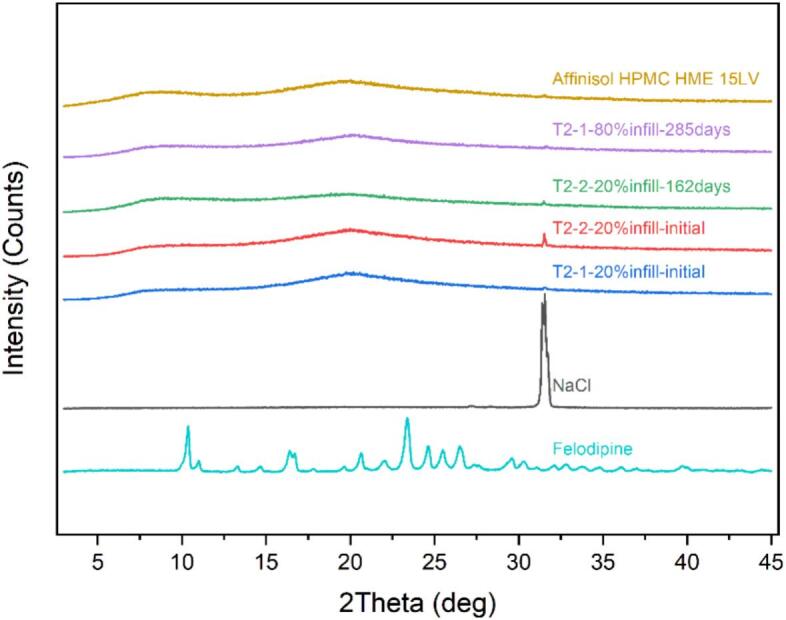
XRD summary of felodipine, NaCl, tablet T2–1-20 %at 0 d, T2–2-20 % at 0 d, T2–2-20 % at 162 d and T2–1-20 % at 285 d in 40 °C/75 %RH storage condition, AffinisolTM HPMC HME 15LV (from bottom to top).

As shown in Fig. 7, the HPMC polymer and 3D printed tablets all exhibit the characteristic peak of NaCl at 2θ of ∼31° in their XRD patterns. This could result from HPMC manufacturing, since usually a sodium hydroxide water solution is added into the raw material cotton wool for alkaline treatment. NaCl could potentially remain as a residue if any chloride-containing materials are added into the production process. Here, we chose to pre-treat the samples with water before conducting PLM characterization. This approach allowed the NaCl residue to dissolve in the HPMC based gel, whilst the drug's crystallinity remained intact in the sample due to its low solubility in water. The risk of the amorphous sample being solubilized in water to change to crystalline has been evaluated at 2, 5 and 10 min, and no form change within these timepoints was observed. To minimize the risk of form change, the PLM characterization is recommended to be done within 2 min after sample pre-treatment.

#### 3.4.3 Physical stability characterization

**Fig. 8:**
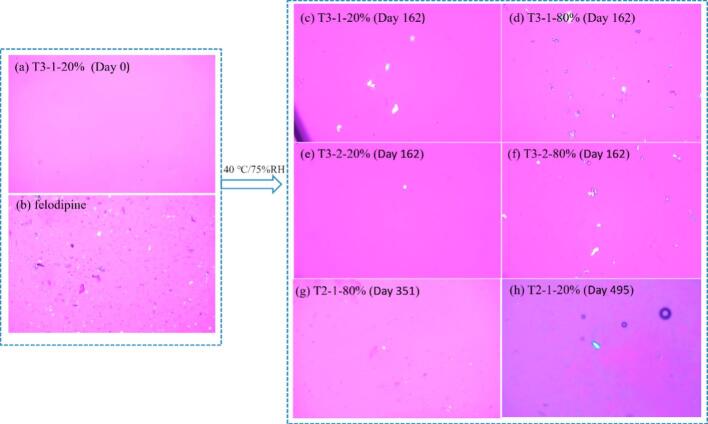
PLM micrographs (40× magnification) of printed tablets under 40 °C/75 %RH storage conditions (a) tablet T3–1-20 % at timepoint 0 d as control (b) crystalline felodipine (c -d) tablet T3–1-20 %, T3–1-80 %, T3–2-20 % and T3–2-80 % at timepoint 162 d (f) tablet T2–1-80 % at timepoint 351 d (g) tablet T2–1-20 % at timepoint 495 d.

For physical stability, all tablets were taken out at different timepoints to be characterized by XRD, DSC and PLM. Under the condition of 25 °C/5 %RH, no physical changes were detected in the tablets. Fig. 8 provides a summary of the tablets that initially showed traces of birefringence using PLM characterization when subjected to conditions of 40 °C/75 %RH. Traces of birefringence in tablets T3–1-20 %, T3–1-80 %, T3–2-20 % and T3–2-80 %, all of which had a ratio FEL-HPMC (w/w 3:7), were firstly observed under PLM after 162 d. The results indicated that FEL-HPMC weight ratio (or drug loads) would be a key parameter for physical stability studies. With higher drug loading, the observed physical stability of samples is less.

**Fig. 9:**
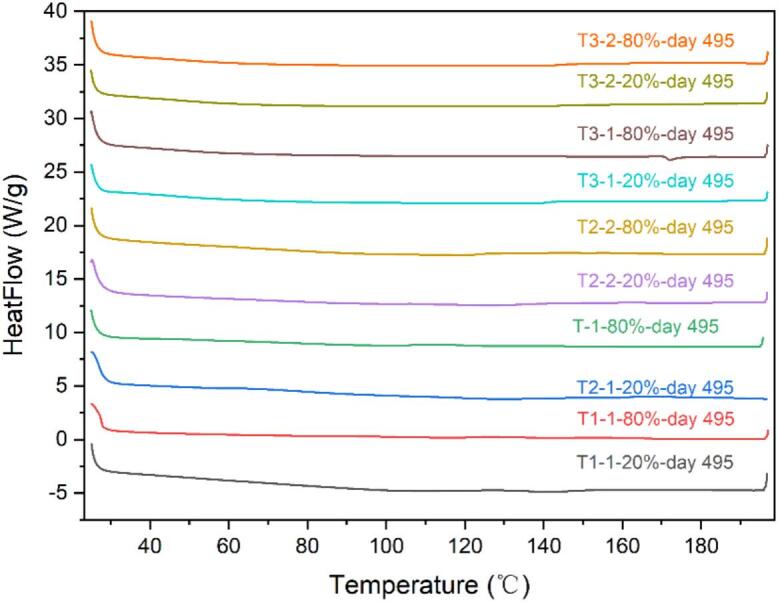
DSC summary of printed tablets under 40 °C/75 %RH storage condition.

**Fig. 10:**
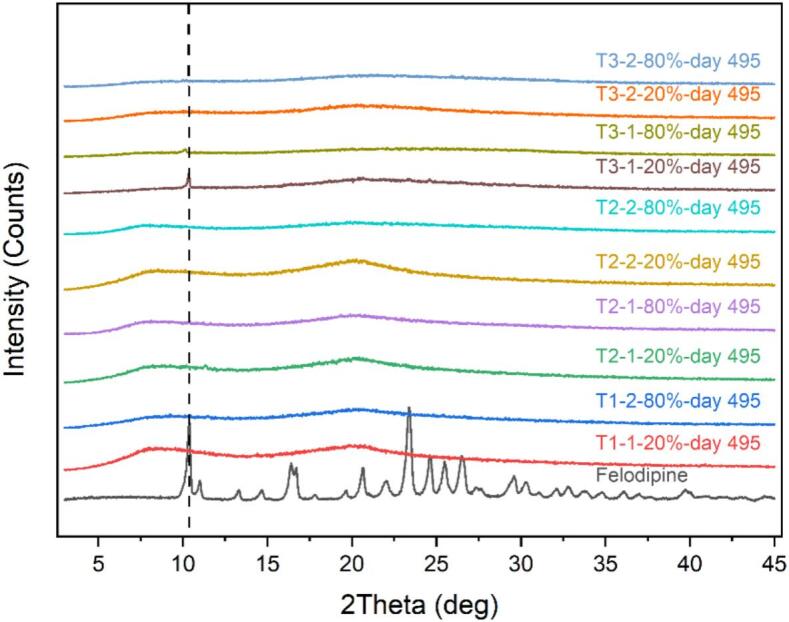
XRD summary of printed tablets under 40 °C/75 %RH storage condition (a) XRD summary of printed tablets at timepoint 495 d (b) from top to bottom: tablet F3–1-80 % at timepoint 495 d, tablet F3–1-20 % at timepoint 495 d, crystalline felodipine.

Although traces of birefringence have been observed in some samples, DSC did not detect any recrystallizations in most samples (Fig. 9), except for tablet T3–1-80 %. Peaks in the XRD patterns for tablets T3–1-20 % and T3–1-80 % could only be observed until 495 days. Additionally, the endotherm peak observed in DSC is at 172.17 °C for Tablet F3–1-80 %, which is higher than the melting point of felodipine (around 145 °C). Further experiments are required to investigate the origin of this new peak, which could be caused by polymorphism of drug, degradation product, etc. The recrystallization of the drug was confirmed by XRD peaks comparison with crystalline felodipine in Fig. 10. Despite the detection of birefringence traces in tablets T3–2-20 % and T3–2-80 % on 162 days, no peaks were observed in the XRD patterns even after 495 days under 40 °C/75 %RH conditions. Hence, we can confirm that the tablets T3–1-20 % and T3–1-80 % showed less stability than T3–2-20 % and T3–2-80 %. Compared with T3–2-20 % and T3–2-80 %, tablets T3–1-80 % and T3–1-20 % were prepared with lower processing temperatures in HME and 3D printing, which indicated that a higher processing temperature may be another key parameter for better physical stability. Naturally, temperature selection must consider its potential impact on extrudability, printability and chemical stability. Higher temperatures may lead to chemical degradation, and sometimes the selected materials may behave different properties (such as viscosity) at different temperatures, so these factors must all be considered in the selection process.

In Fig. 8, birefringence traces for tablet T2–1-80 % were firstly observed at 351 days, while for tablet T2–1-20 %, they were observed at 495 days, and hence in the context of crystallisation from the amorphous state is the more physically stable of the two. However, both are less stable than tablets processed at higher HME and 3D printing temperatures (T2–2-20 % and T2–2-80 %), and more stable than tablets with higher drug loads (30 %, *w*/w), with a FEL-HMPC weight ratio 3:7. These results further provide evidence that lower drug loads and elevated processing temperatures have a positive impact on the physical stability of the printed tablets. For the tablet T2–1-20 % and T2–1-80 %, the main difference lies in infill density used during the 3D printing process. It has been observed that, with a higher infill density potentially adversely affects affecting the physical stability of the tablets. This result is consistent with the DSC data shown in Fig. 9, where an endothermic peak was observed in the DSC curve of T3–1-80 % at the 495-day timepoint, but not in the case of tablet T3–1-20 %. As can be seen clearly in Fig. S1 all printed tablets present a solid surface regardless of infill density and very similar volumes. Hence surface area to volume ratios remain approximately constant and is an unlikely factor in physical stability in relation to water absorption. There are, however, clear differences in density with T3–1-80 % having a density of 1.03 g/cm3 and T3–1-20 % having a density of 0.68 g cm^−3^ (Table S2). The improved observed physical stability of the less dense and hence lighter 20 % infill tablet is therefore likely due to a lower drug content (and spatial density) making nucleation and crystallisation less likely.

The influence of drug loads and elevated processing temperature on physical stability aligns with the findings regarding the variation of Gibb's free energy of mixing and the phase diagram. As depicted in Fig. 4, the ΔG_mix_ begins to decrease and becomes increasingly negative as the temperature rises. This suggests the creation of more uniform mixtures, potentially leading to improved physical stability. The phase diagram in Fig. 5 illustrates that the system with a 10 % (*w*/w) drug load at 45 °C is in a metastable state and begins to shift towards the unstable region as the drug load increases. This could be due to reduced miscibility with increased drug loads, as the drug concentration nears saturation of the polymer.

Affinisol™ HPMC HME 15LV is an HPMC polymer with varying substituents that exhibits a degree of hygroscopicity (e.g., up to ∼20 % water uptake by weight from 0 % to 100 % relative humidity). When considering the effect of infill density on the physical stability of printed tablets, a lower infill density may result in tablets of less water content absorption due to fewer polymers or materials within the same matrix volume. Variations in water content during storage could gradually modify the polymer structure (for example, by reducing Tg and enhancing drug mobility), which could potentially undermine the physical stability of the drug product (Xiang and Anderson, 2017). However, it is important to note that the water content in tablets with the lower infill density tablets could be higher than that of tablets with higher infill density if the water has not reached equilibrium within the tablet and may have negative impact on physical stability.

## 4 Conclusions

In conclusion, this study has developed a structured and efficient method for boosting felodipine-HPMC based FDM 3D formulation and printing by facilitating the better and quicker selection of material ratios and processing parameters. The integration of rheological studies, theoretical analysis, and phase diagram construction has enabled the possibility to predict the extrudability and printability of materials and select suitable temperature conditions, which accelerate the FDM printing process. Our findings demonstrate that lower drug loading and elevated processing temperatures can enhance the physical stability of printed tablets, while lower infill density may compromise stability due to a lower drug content (and spatial density) making nucleation and crystallisation less likely. These insights offer practical guidance for optimizing the formulation and processing of FDM 3D printed products.

Although this study focused on a single drug-polymer system, the methodology is versatile and can be adapted to other materials, paving the way for broader applications in FDM 3D printing. With further data and refinement, this approach has the potential to significantly improve the quality and stability of 3D printed medicinal products. We believe these findings contribute to the ongoing development of FDM 3D printing as a robust and reliable technology for pharmaceutical manufacturing.

## CRediT authorship contribution statement

**Haixia Ren:** Writing – original draft, Methodology, Investigation, Formal analysis, Data curation. **Charles A. Laughton:** Writing – review & editing, Supervision, Conceptualization. **Clive J. Roberts:** Writing – review & editing, Supervision, Conceptualization. **Kam Loon Fow:** Writing – review & editing, Supervision, Resources, Funding acquisition, Conceptualization.

## Declaration of competing interest

The authors declare the following financial interests/personal relationships which may be considered as potential competing interests:

Kam Loon Fow reports financial support was provided by 10.13039/100007834Ningbo Municipal Natural Science Foundation. If there are other authors, they declare that they have no known competing financial interests or personal relationships that could have appeared to influence the work reported in this paper.

## Data Availability

Data will be made available on request.

## Appendix A Supplementary data

Supplementary material Image 1
