# Supplementary material for: Development of an efficient approach to boost fused deposition modeling (FDM) printing of felodipine-HPMC tablets for enhanced physical stability
Source: Int J Pharm X. 2025 Sep 12;10:100394. doi: 10.1016/j.ijpx.2025.100394 (PMC12538912; doi:10.1016/j.ijpx.2025.100394)
Supplement: Supplementary file 1 — Supplementary material [file mmc1.docx]

**Development of an Efficient Approach to Boost Fused Deposition Modeling (FDM) Printing of Felodipine-HPMC Tablets for Enhanced Physical Stability**

Haixia Ren^a^, Charles A Laughton^b^, Clive J Roberts^c*^, Kam Loon Fow^a*^

^a^ Department of Chemical and Environmental Engineering, University of Nottingham Ningbo China, Ningbo 315100, China

^b^ School of Pharmacy, University of Nottingham, Nottingham NG7 2RD, UK

^c^ School of Life Sciences, University of Nottingham, Nottingham NG7 2UH, UK

* Corresponding Authors.

E-mail addresses: clive.roberts@nottingham.ac.uk; kam-loon.fow@nottingham.edu.cn

### **Supplementary Material _ Filaments and Tablets Data**

*1.1. Filaments results*

To determine the average diameter, a filament for each formulation was randomly selected. The diameter of filaments was measured at 5 points using a vernier caliper. Average and standard deviation (std) of diameter were calculated accordingly. Detailed information is summarized in **Table S1** including photos. For filaments F4-1 and F4-2, which contain FEL-HPMC (w/w 50:50), the diameter could not be consistently controlled, making them unsuitable for feeding into the subsequent FDM printer.

**Table S1**: Diameter and pictures of fabricated filaments

| **Filament** | **Filament diameter**  **± SD (mm)** | **Pictures** |
| --- | --- | --- |
| F1-1 | 1.71 ± 0.04 (n=5) | 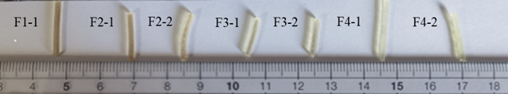 |
| F1-2 | 1.72 ± 0.04 (n=5) |  |
| F2-1 | 1.70 ± 0.04 (n=5) |  |
| F2-2 | 1.73 ± 0.03 (n=5) |  |
| F3-1 | 1.72 ± 0.03 (n=5) |  |
| F3-2 | 1.70 ± 0.03 (n=5) |  |
| F4-1 | n.a* |  |
| F4-2 | n.a* |  |

*: The diameter variability is high (e.g. less than 1.55 mm or higher than 1.80 mm)

*1.2. Tablets results*

10 tablets were calculated for tablet size, weight and density. Results were summarized in **Table S2.**

**Table S2:** FDM tablets size and weight

| **Tablet Name** | **Diameter**  **± SD ((mm)** | **Thickness**  **± SD ((mm)** | **Weight**  **± SD (mg)** | **Density (g/cm3)** |
| --- | --- | --- | --- | --- |
| T1-1-20% | 6.22± 0.22 | 2.41± 0.08 | 46.52 ± 4.30 | 0.64 |
| T1-2-80% | 6.12± 0.21 | 2.28± 0.07 | 59.99 ± 6.39 | 0.89 |
| T2-1-20% | 6.03± 0.12 | 2.46± 0.06 | 43.80 ± 1.23 | 0.62 |
| T2-1-80% | 5.95± 0.08 | 2.49± 0.09 | 66.31 ± 0.96 | 0.96 |
| T2-2-20% | 6.21± 0.13 | 2.15± 0.04 | 47.00 ± 2.22 | 0.72 |
| T2-2-80% | 6.05± 0.15 | 2.54± 0.20 | 68.64 ± 5.60 | 0.94 |
| T3-1-20% | 6.00± 0.11 | 2.52± 0.08 | 48.32 ± 2.62 | 0.68 |
| T3-1-80% | 6.11± 0.10 | 2.49± 0.10 | 75.27 ± 2.91 | 1.03 |
| T3-2-20% | 6.25± 0.14 | 2.58± 0.07 | 53.68 ± 2.49 | 0.68 |
| T3-2-80% | 6.11± 0.21 | 2.59± 0.07 | 75.57 ± 2.23 | 1.00 |

The appearance of all printed tablets is smooth, and the surface is intact with no damage (**Fig. S1**). Their color varies from light to dull pale yellow, tending to become duller pale yellow as drug loading decreases or processing temperature increases.


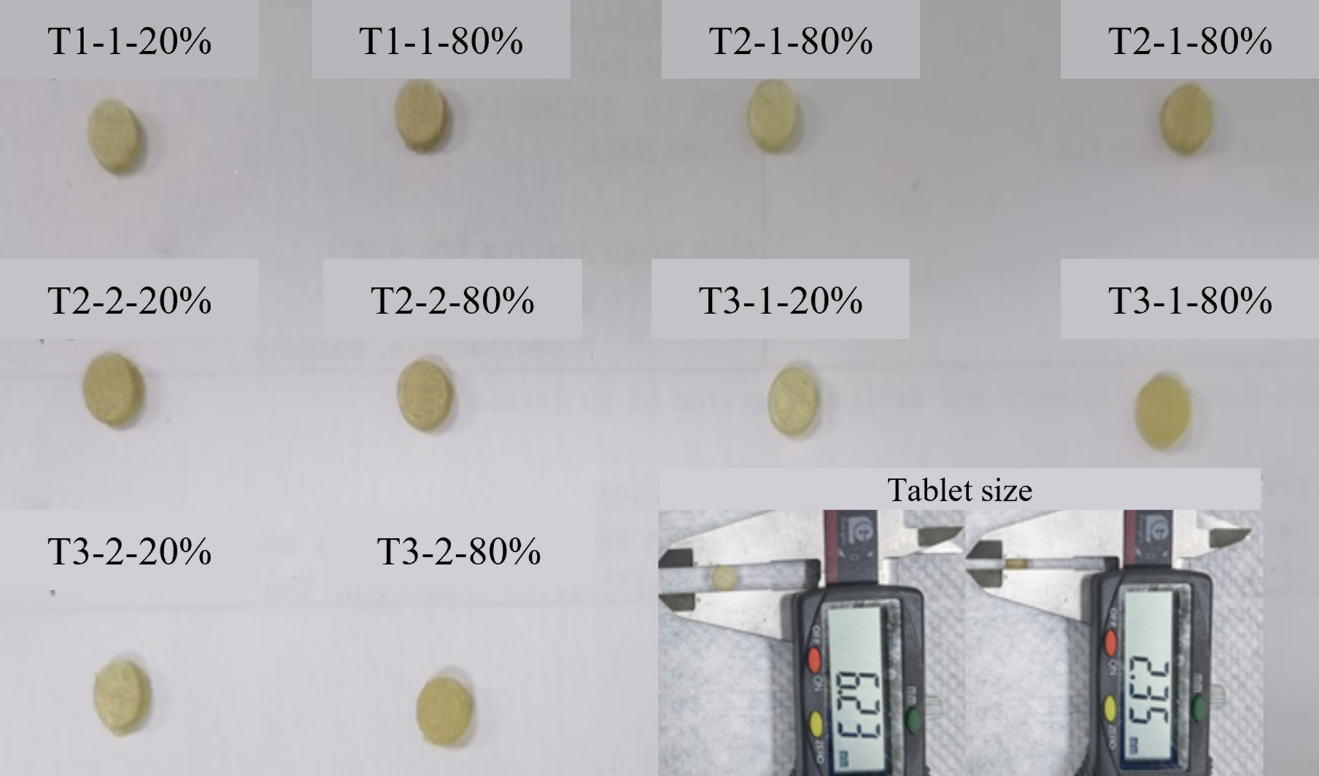


**Fig. S1** Tablets pictures based on different formulation and process parameters

### **Supplementary Material _ Screw Configuration**

The screw configuration of the hot melt extruder used for filament fabrication is displayed in **Fig. S2.**


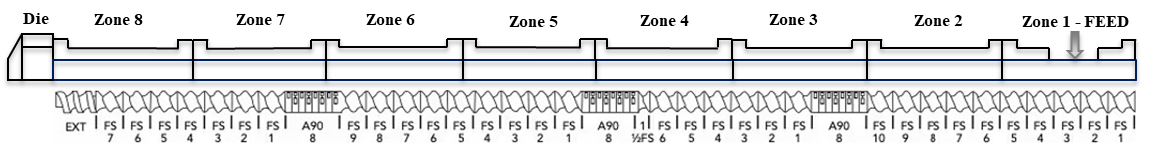


**Fig. S2** Screw configuration for hot melt extrusion

### **Supplementary Material _ PLM Micrographs of printed tablets under different timepoints at 40 ºC/75%RH storage conditions**


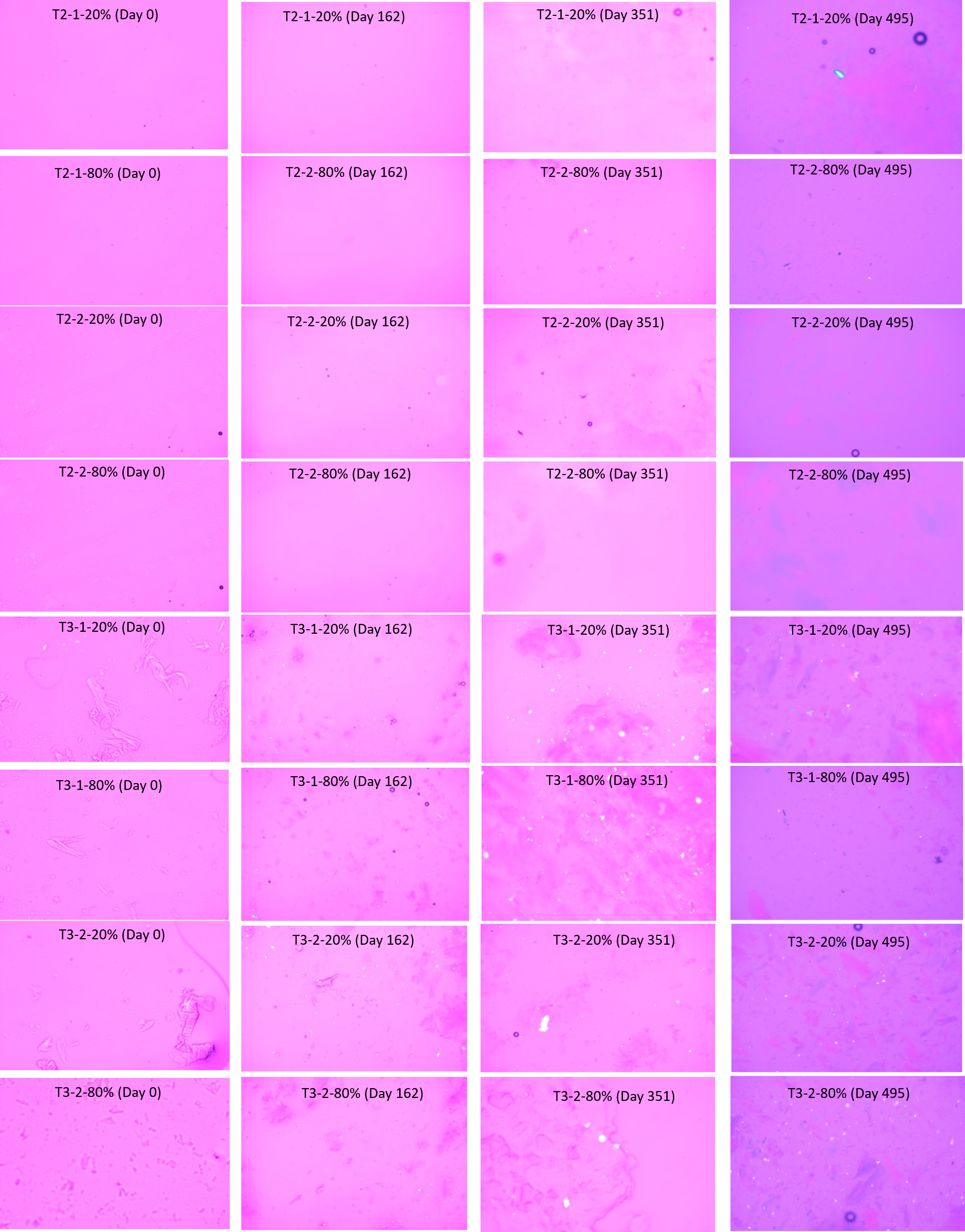


**Fig. S3** PLM micrographs of printed tablets under 40 ºC/75%RH storage conditions. From left to right: PLM micrographs under different time point (day 0, day 162, day 351 and day 495). From top to bottom: PLM micrographs with different tablets
